# Acute resistance exercise training does not augment mitochondrial remodelling in master athletes or untrained older adults

**DOI:** 10.3389/fphys.2022.1097988

**Published:** 2023-01-04

**Authors:** Ryan Neil Marshall, James McKendry, Benoit Smeuninx, Alex Peter Seabright, Paul T. Morgan, Carolyn Greig, Leigh Breen

**Affiliations:** ^1^ School of Sport, Exercise, and Rehabilitation Sciences, University of Birmingham, Birmingham, United Kingdom; ^2^ MRC-Versus Arthritis Centre for Musculoskeletal Ageing Research, Birmingham, United Kingdom; ^3^ Exercise Metabolism Research Group, Department of Kinesiology, McMaster University, Hamilton, ON, Canada; ^4^ NIHR Biomedical Research Centre, Birmingham, United Kingdom

**Keywords:** ageing, skeletal muscle, mitochondria, resistance exercise, healthy ageing

## Abstract

**Background:** Ageing is associated with alterations to skeletal muscle oxidative metabolism that may be influenced by physical activity status, although the mechanisms underlying these changes have not been unraveled. Similarly, the effect of resistance exercise training (RET) on skeletal muscle mitochondrial regulation is unclear.

**Methods:** Seven endurance-trained masters athletes ([MA], 74 ± 3 years) and seven untrained older adults ([OC]. 69 ± 6 years) completed a single session of knee extension RET (6 x 12 repetitions, 75% 1-RM, 120-s intra-set recovery). Vastus lateralis muscle biopsies were collected pre-RET, 1 h post-RET, and 48h post-RET. Skeletal muscle biopsies were analyzed for citrate synthase (CS) enzyme activity, mitochondrial content, and markers of mitochondrial quality control *via* immunoblotting.

**Results:** Pre-RET CS activity and protein content were ∼45% (*p* < .001) and ∼74% greater in MA compared with OC (*p* = .006). There was a significant reduction (∼18%) in CS activity 48 h post-RET (*p* < .05) in OC, but not MA. Pre-RET abundance of individual and combined mitochondrial electron transport chain (ETC) complexes I-V were significantly greater in MA compared with OC, as were markers of mitochondrial fission and fusion dynamics (p-DRP-1^Ser616^, p-MFF^Ser146^, OPA-1 & FIS-1, *p* < .05 for all). Moreover, MA displayed greater expression of p-AMPK^Thr172^, PGC1α, TFAM, and SIRT-3 (*p* < .05 for all). Notably, RET did not alter the expression of any marker of mitochondrial content, biogenesis, or quality control in both OC and MA.

**Conclusion:** The present data suggest that long-term aerobic exercise training supports superior skeletal muscle mitochondrial density and protein content into later life, which may be regulated by greater mitochondrial quality control mechanisms and supported *via* superior fission-fusion dynamics. However, a single session of RET is unable to induce mitochondrial remodelling in the acute (1h post-RET) and delayed (48 h post-RET) recovery period in OC and MA.

## 1 Introduction

The number of older adults (>65 years of age) is growing, and predictions indicate that the worldwide population of this sub-group of adults will reach >2 billion by 2050 (McKinney, 2011). This demographic shift presents a substantial burden for healthcare providers and national healthcare expenditure (Pinedo-Villanueva et al., 2019). Ageing is associated with a gradual decline in skeletal muscle mass and function, termed “sarcopenia” (Cruz-Jentoft et al., 2019). The loss of skeletal muscle mass proceeds at a rate of 1%–2% per year, whereas muscle strength is lost more rapidly (1%–5% per year) (Keller and Engelhardt, 2013). In parallel with the declines in skeletal muscle mass and function, there is also an age-related deterioration in mitochondrial function at a rate of ∼8% per decade (Short et al., 2005a). Furthermore, poor mitochondrial energetics has been associated with greater functional impairment in day-to-day tasks, resulting in more severe declines in mobility across the lifespan (Gonzalez-Freire et al., 2018). However, participating in regular physical activity and exercise throughout adult life may preserve or at least attenuate the rate of decline in muscle mass, function, and cardiorespiratory fitness (Mckendry et al., 2018), with implications for extending healthspan and minimizing years spent in poor health (Seals et al., 2016).

Older adults who have maintained structured endurance exercise training over an extended period of their life (termed “Master Athletes” [MA], lifelong exercisers and/or high-functioning older adults) display consistently greater indices of whole-body and tissue-specific health and exhibit similar metabolic and phenotypic profiles to healthy younger adults (YA) (Pollock et al., 2015; Pollock et al., 2018; Mckendry et al., 2018). As higher mitochondrial content and oxidative capacity have been implicated in a lower disease prevalence (Short et al., 2005b), undertaking endurance exercise training across the lifespan may offset age-related mitochondrial dysfunction (Ringholm et al., 2022). Indeed, compared with healthy age-matched adults, MA display superior mitochondrial function, content, biogenesis, and quality control (Zampieri et al., 2015; Pollock et al., 2018; Gudiksen et al., 2022; Ringholm et al., 2022; Ubaida-Mohien et al., 2022). Therefore, the maintenance of physical activity and structured exercise training across the lifespan may contribute to the attenuation of mitochondrial function and muscle health decline with ageing.

The single most potent strategy to maintain skeletal muscle mass, strength, and function in older age is resistance exercise training (RET) (Peterson et al., 2010; Morton et al., 2018). However, to date, research investigating RET-induced molecular signalling and adaptative remodelling in YA and older adults (OA) has primarily centred around the muscle anabolic role of mTORC1 (mechanistic target of rapamycin complex 1), the acute elevations in muscle protein synthesis, and the long-term accrual of lean muscle mass (Joanisse et al., 2020a). Since seminal observations on RET-induced mitochondrial adaptations in older adults (Jubrias et al., 2001; Melov et al., 2007; Robinson et al., 2017b), recent advances have begun to evaluate the mechanistic and molecular underpinnings of how RET augments mitochondrial remodelling utilizing acute and chronic exercise training models (Mesquita et al., 2020; Mesquita et al., 2021). Mitochondria undergo dynamic remodelling *via* biosynthesis, fission, fusion, and degradation of dysfunctional organelles (Drake et al., 2016). Given that RET may be beneficial to facilitate the simultaneous accrual of myofibrillar and mitochondrial proteins, with concurrent improvements in mitochondrial function (Porter et al., 2015a; Groennebaek et al., 2018a; Parry et al., 2020; Mesquita et al., 2021), understanding how acute RET modulates the mitochondrial regulatory signalling pathways is imperative for tailored exercise prescription to improve the musculoskeletal and general health of older adults. However, our understating of how RET augments mitochondrial remodelling in lifelong exercisers remains unanswered.

Therefore, the first aim of the present study was to comprehensively assess molecular signalling regulating skeletal muscle mitochondrial and substrate metabolism (i.e., content, fission-fusion, and biogenesis) in endurance-trained MA compared with untrained healthy age-matched older adults (OC). Secondly, we aimed to determine the effect of a single bout of RET on muscle mitochondrial-mediated signalling in the early (1 h) and late-phase (48 h) of recovery in MA and OC. We hypothesized that endurance-trained MA would display a greater abundance and activity of mitochondrial protein expression. Secondly, we hypothesized RET would increase post-RET mitochondrial remodelling *via* protein phosphorylation in both cohorts of participants, with a blunted response in MA.

## 2 Materials and Methods

### 2.1 Ethical approval

The present study was a retrospective analysis of skeletal muscle biopsy samples obtained as part of a larger trial on the effects of RET on integrated myofibrillar protein synthesis between endurance-trained master athletes and untrained older individuals (McKendry et al., 2019). Briefly, prior to participation in the study, written informed consent was obtained from all subjects. All procedures were approved by the National Health Service East Midlands Research Ethics Committee (18/EM/0004) and the University of Birmingham Governance and Research Ethics Committee (RG_17–187).

### 2.2 Participants

Seven untrained older males (OC; 74) ± 3 years, BMI; 24.8 ± 3.3 kg/m^−2^) and seven endurance-trained master athletes (MA; 69 ± 6 years, BMI; 22.0 ± 1.6 kg/m^−2^) were recruited as previously described (McKendry et al., 2019). Briefly, OC were deemed eligible for participation if they were habitually physically active (i.e., >7,000 steps/day^−1^) and had not previously participated in or followed any structured exercise training. MA were eligible if they had maintained continuous endurance exercise training at least twice per week for >20 years preceding the study. The endurance training history of MA has been reported previously (exercise experience; 48 ± 15 years, sessions per week; 4.3 ± 1.6, training volume; 8.4 ± 6.6 h) (McKendry et al., 2019).

### 2.3 Study design

In a parallel study design, as previously described (McKendry et al., 2019), OC and MA were recruited to determine the acute (1 h) and delayed (48 h) post-exercise molecular signalling regulating skeletal muscle mitochondrial quality control. Participants reported to The School of Sport, Exercise and Rehabilitation Sciences (University of Birmingham) in an overnight fasted state. A single skeletal muscle biopsy was collected upon arrival, followed by the completion of a single bout of knee extension RET (6 x 12 repetitions, 75% 1-RM, 120-s intra-set recovery). Muscle biopsy samples were obtained from the quadriceps *vastus lateralis* under local anaesthesia (1% lidocaine) using the Bergström needle technique. Muscle biopsy tissue was quickly rinsed in ice-cold saline and blotted to remove any visible fat and connective tissue before being frozen in liquid nitrogen. Following cessation of RET, participants rested for 1 h before another muscle biopsy was sampled from the same leg. Finally, 48 h post-exercise, participants returned to the laboratory following an overnight fast for further muscle biopsy sampling ∼3 cm proximal to the previous sampling site. RET was selected as the exercise mode as both OC and MA would be largely unaccustomed to the nature of the contractile stimulus.

### 2.4 Immunoblotting

Immunoblotting was performed on the sarcoplasmic fraction of skeletal muscle biopsy samples that remained following myofibrillar protein extraction, as previously reported (McKendry et al., 2019). Briefly, 30–35 mg of snap-frozen vastus lateralis skeletal muscle was mechanically homogenized using a TissueLyser II- and 5-mm stainless steel beads (Qiagen, Hannover, Germany). Samples were disrupted following 3 x 2-min cycles at 20 Hz in ice-cold homogenization buffer (25 mM Tris buffer [Tris–HCl, Trizma Base, 25 mL of Milli-Q H_2_O, pH 7.2], 100 μL TritonX-100, one PhosSTOP Tablet (Roche, Switzerland), one complete protease inhibitor tab) (Roche, West Sussex, United Kingdom) at 10 μL/μg per tissue and shaken on a vibrax shaker at 4°C for 10 min. Homogenates were spun at 4,500 g for 10 min at 4°C, and the sarcoplasmic supernatant was collected and frozen at −80°C for subsequent western blot analysis. Protein content was determined by DC Protein Assay (Bio-Rad, Hertfordshire, United Kingdom). Western blot aliquots were prepared at 3μg/1 μL in 4 x Laemmli sample buffer and ddH_2_O. Following preparation, samples were left at room temperature for 24 h to denature. Equal amounts of protein (30 μg) were loaded into 4%–15% Tris-Glycine precast gels (BioRad, Hertfordshire, United Kingdom) and separated by SDS-PAGE for 1 h at 140 V in pre-made tris-glycine running buffer (24 mM Tris, 192 mM *Glycine*, pH 8.3). Proteins were transferred to a polyvinylidene difluoride (PVDF; Whatman, Dassel, Germany) membrane at 100 V for 1 h in a pre-made transfer buffer. Membranes were blocked in 5% low-fat milk (diluted in Tris-buffered saline and .1% Tween-20 (TBS-T)) for 1 h at room temperature. The membranes were incubated overnight (4°C) with appropriate antibodies (see “Antibodies”). Following overnight incubation, membranes were washed 3 × 5 min in TBS-T, incubated in horseradish peroxidase (Mouse/Rabbit-HRP)-conjugated secondary antibody (Cell Signaling, 7076/7074, 1:10,000 in 2.5% BSA in TBS-T) at room temperature for 1 h, before the last 3 × 5 min washes in TBS-T. Membranes were exposed to Chemiluminescent HRP Substrate (Millipore Corp, Billerica, MA, United States) for 2-min and bands were visualized by a Chemi XT4 imager with GeneSys capture software (Syngene UK, Cambridge, UK). Quantification was performed using ImageJ/Fiji (NIH, Bethesda, MD, United States). Relative arbitrary units were normalized to the total amount of protein loaded as visualized *via* Ponceau S staining (Romero-Calvo et al., 2010). Specifically, bands were normalized to the ∼45 kDa actin band of the Ponceau S stain as a loading control (Gilda and Gomes, 2013). No difference was detected between the ∼45 kDa actin band of the Ponceau S stain across all samples. Following these corrections, where appropriate, the phosphorylation of proteins as a proxy of their activation is expressed relative to each protein’s total abundance (Bass et al., 2017).

### 2.5 Antibodies

All primary antibodies were prepared in TBST. Antibodies were as follows; total OXPHOS human antibody cocktail (Manufacturer; Abcam, Catalogue Number; 110411, diluted 1:1000 in tris-buffered saline with .1% Tween 20 (TBST), Citrate Synthase (CS; Cell Signaling Technology [CST], 14309, 1:1000), Mitofusin-2 (MFN2; CST, 9482, 1:1000), Dynamin-Related Protein-1 (DRP-1; CST, 8570, 1:1000), Phosphorylated DRP-1^Ser616^ (p-DRP-1^Ser616^, CST, 4494, 1:1,000), Optic Atrophy-1 (OPA-1; BD Bioscience, 612607, 1:1000), Mitochondrial Fission Factor (MFF; CST, 84580, 1:1000), Phosphorylated Mitochondrial Fission Factor (p-MFF^Ser146^; CST, 49281, 1:1000), Mitochondrial Fission Protein-1 (FIS-1; Atlas Antibodies, HPA017430, 1:1000), Mitofilin (ProteinTech, 10179-AP, 1:1000), Total AMP-activated protein kinase, (AMPK; CST, 2532, 1:1000), Phosphorylated AMPK^T172^ (p-AMPK^Thr172^, CST, 2535, 1:1000), Total Acetyl-CoA carboxylase (ACC; Dundee MRC DSTT, N/A [custom made], 1:1000), Phosphorylated ACC^Ser79^ (p-ACC^Ser79^, Dundee MRC DSTT, N/A [custom made], 1:1000), Peroxisome proliferator-activated receptor gamma coactivator 1-alpha (PGC-1α; Abcam, 3242, 1:1000), mitochondrial transcription factor A (TFAM; Sigma-Aldrich, SAB1401383, 1:1000), Sirtuin-3 (SIRT3; CST, D22A3, 1:1000), Poly-ADP Ribose Polymerase 1 (PARP1; CST, 9542, 1:1000), Nicotinamide Riboside kinase 1/2 (NMRK1 and NMRK2; BioGenes, Berlin, Germany, N/A [custom made], 1:1000). Appropriate (Mouse/Rabbit) HRP conjugated secondary antibody (CST, 7076 & 7074, 1:10,000 in 2.5% BSA in TBS-T) following overnight incubations.

### 2.6 Enzyme activity

Citrate Synthase (CS) enzyme activity was determined following the protocols of Spinazzi et al. (Spinazzi et al., 2012) and adapted to 96-well plate format as previously described (Janssen and Boyle, 2019). In brief, 20–30 mg of skeletal muscle was homogenized in 10 μL/μg of ice-cool sucrose lysis buffer (50 mM Tris, 1 mM EDTA, 1 mM EGTA, 50 mM NaF, 5 mM Na4P2O7-10H2O, 270 mM sucrose, 1 M Triton-X, 25 mM *β*-glycerophosphate, 1 μM Trichostatin A, 10 mM Nicotinamide, 1 mM 1,4-Dithiothreitol, 1% Phosphatase Inhibitor Cocktail 2; Sigma, 1% Sigma Phosphatase Inhibitor Cocktail 2; Sigma, 4.8% complete Mini Protease Inhibitor Cocktail; Roche) using a TissueLyser II, and 5 mm stainless steel beads (Qiagen, Hannover, Germany). Samples were disrupted following 3 x 2-min cycles at 20 Hz, followed by centrifugation at 13,000 RPM for 10 min. The protein content of the resulting supernatant was determined *via* DC protein assay (Bio-Rad, Hercules, California, United States) and prepared at 2 μg/μL in ddH_2_O. For determination of CS activity, 10 μL of sample (total of 20 μg of protein) was diluted in 186 μL of assay solution (50 mM Tris buffer (pH 7.4) with 100 μM DTNB, 115 μM acetyl-CoA in ddH_2_O) to a total well volume of 196 μL, as previously described (Janssen and Boyle, 2019). Baseline absorbance was read every 15 s for 3 minutes in a microplate reader (FLUOstar Omega, BMG Labtech, Aylesbury, United Kingdom). Following the baseline reading, 4 μL of 5 mM oxaloacetate (100 μM final concentration) was added to start the reaction and absorbance was read again every 15 s for 3 minutes. CS activity was calculated using the extinction coefficient (CS *ε* = 13.6). Measurement CV% within-plate was 6.6 ± 1.7% for three technical repeats (Kuang et al., 2021).

### 2.7 Statistical analysis

Enzyme activity and immunoblotting data were compared using a Two-Way ANOVA with a between-group factor (Group [OC vs. MA]) and within-group factor (Time [0 h vs. 1 h vs. 48 h post-RET). Bonferroni *post hoc* analysis was applied to correct for multiple comparisons. Significance was set at *p* < .05. Data are presented as mean ± SEM unless otherwise stated, and displayed as fold-change relative to the pre-exercise OC. All analysis was conducted using GraphPad Software Inc. Prism version 8.

## 3 Results

### 3.1 Enzyme activity and mitochondrial OXPHOS content

**FIGURE 1:**
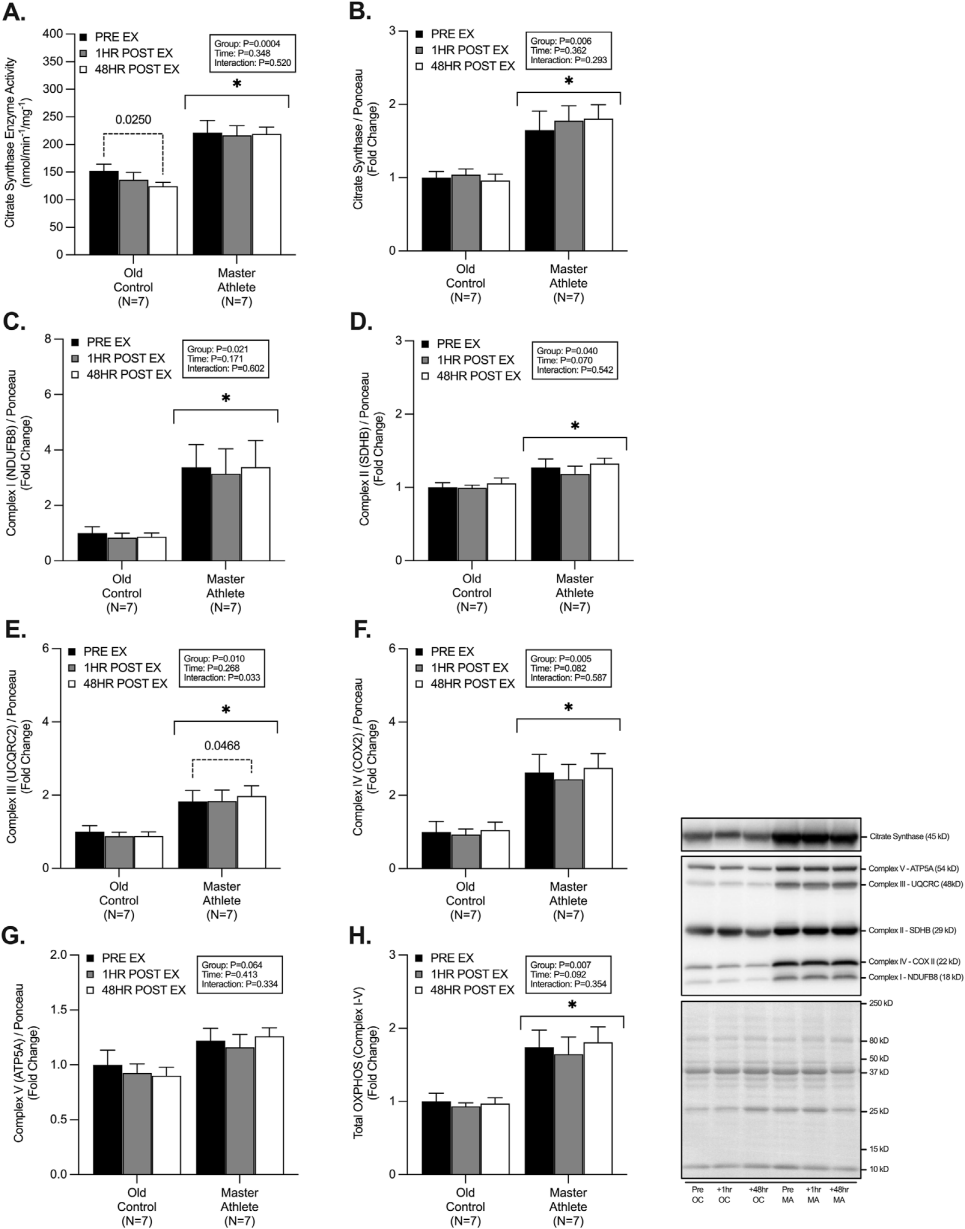
Master athletes display greater citrate synthase enzyme activity and mitochondrial content compared with old controls. Citrate synthase enzyme activity **(A)** citrate synthase protein expression **(B)** and protein expression of electron transport chain complexes I-V; NDUFB8 **(C)**, SDHB **(D)**, UQCRC **(E)**, COX II **(F)**, ATP5A **(G)**, Total OXPHOS **(H)** at rest (Black), 1 h post-resistance exercise (Grey), and 48 h post-resistance exercise (White) in older untrained controls (N = 7) and master athletes (N = 7). All proteins are expressed relative to the total protein stain (Ponceau S). Significance was set at *p* < .05. The symbol “∗” with solid black bars indicates significantly different from older untrained control (*p* < .05). The *p*-value with dotted lines indicates significantly different from pre-exercise rest (*p* < .05). Values are presented as fold-change relative to pre-exercise old control.

CS enzyme activity was ∼65% greater CS enzyme activity in MA (221.5 ± 56.8 nmol/min/mg) compared with OC (152.08 ± 32.6 nmol/min/mg) (*p* < .001). Post hoc analysis also revealed a significant decline (∼18%) in CS enzyme activity 48 h post-exercise in OC compared with pre-RET (pre-RET: 152 nmol/min/mg vs. 48 h post-RET: 124 nmol/min/mg) (*p* = .025) (Figure 1A). CS protein expression was ∼74% greater in MA compared with OC (*p* = .006), with no effect of RET (*p* = .362) (Figure 1B). The protein abundance of electron transport chain (ETC) proteins (I-IV) was significantly greater in MA compared with OC (Complex I [NDUFB8], ∼265%, *p* = .021, Complex II [SDHB], ∼24%, *p* = .040, Complex III [UQCRC2], ∼104%, *p* = .010, Complex IV [COXII], ∼162%, *p* = .005, and Total ETC, ∼80%, *p* = .007), with no differences in Complex V protein abundance (ATP5A, *p* = .064) between groups (Figures 1C–H). No effect of time effect was observed in the 1h or 48h post-RET period for ETC proteins between groups (Complex I, *p* = .171, Complex II, *p* = .070, Complex IV, *p* = .082, Complex V, *p* = .413, Total ETC, *p* = .81) (Figures 1C–H). However, there was a significant time effect of RET in Complex III expression (*p* = .033), with *post hoc* analysis revealing a small (+8%) but significant (*p* = .046) increase in the 48h post-RET in MA only.

### 3.2 Mitochondrial fission and fusion dynamics

**FIGURE 2:**
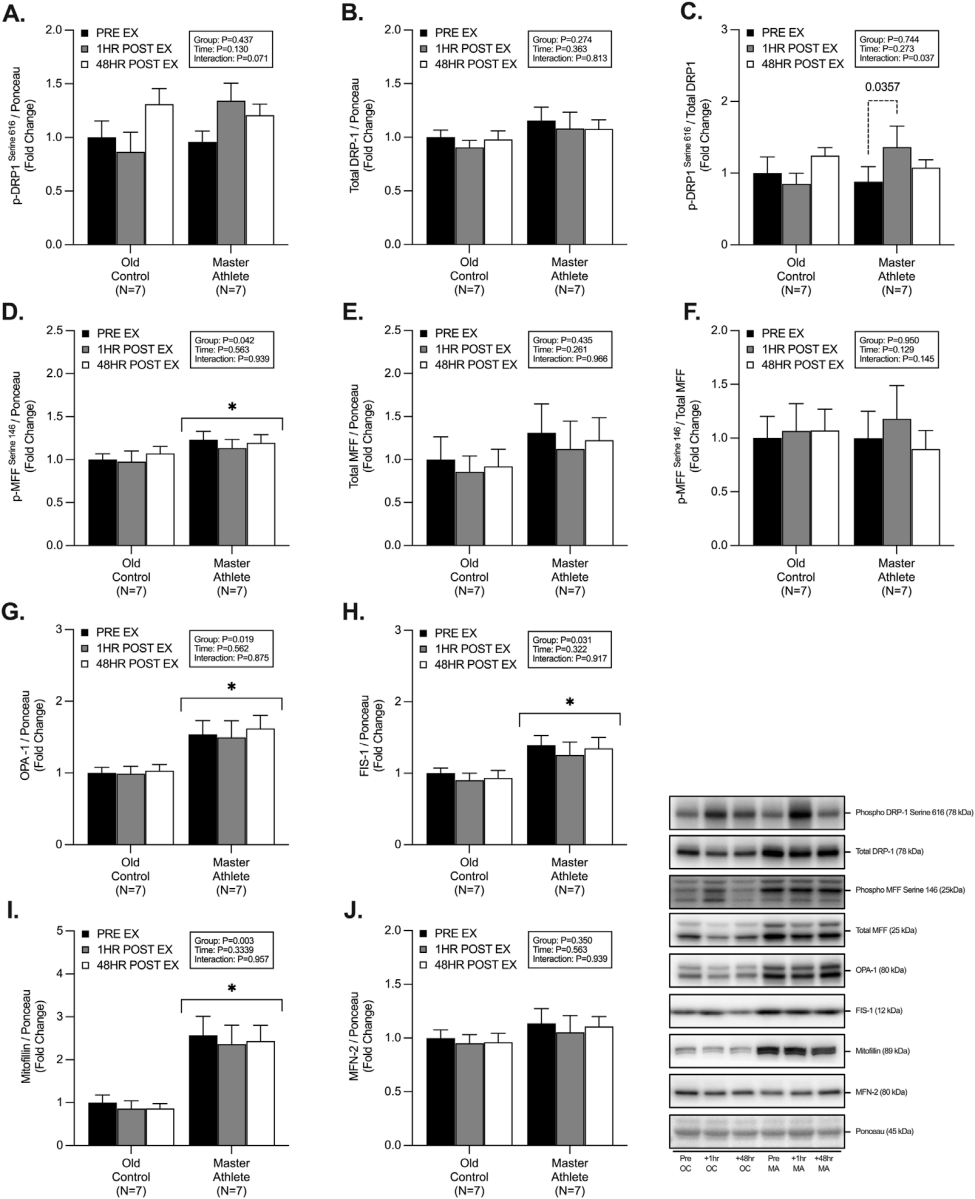
Master athletes display greater abundance of proteins involved in fission and fusion dynamics compared with old controls. Intracellular mitochondrial signalling of phosphorylated DRP1^Serine 616^ **(A)** total DRP1 **(B)** phosphorylated:total DRP1 **(C)**, phosphorylated MFF^Serine 146^ **(D)**, total MFF **(E)**, phosphorylated:total MFF **(F)**, OPA-1 **(G)**, FIS1 **(H)**, Mitofilin **(I)**, MFN-2 **(J)** at rest (Black), 1 h post-resistance exercise (Grey), and 48 h post-resistance exercise (White) in older untrained controls (N = 7) and master athletes (N = 7). All proteins are expressed relative to the total protein stain (Ponceau S) and, where relevant, expressed at phosphorylated:total as a proxy for the respective activation status of the target protein. Significance was set at *p* < .05. The symbol “∗” with solid black bars indicates significantly different from older untrained control (*p* < .05). The *p*-value with dotted lines indicates significantly different from pre-exercise rest (*p* < .05). Values are presented as fold-change relative to pre-exercise old control.

MA displayed significantly greater abundance of; p-MFF^Ser146^ (∼17%, *p* = .042, Figure 2D), OPA-1 (∼55%, *p* = .019, Figure 2G), FIS-1 (∼41%, *p* = .031, Figure 2H) and Mitofilin (∼270%, *p* = .003, Figure 2I). However, we observed no significant differences between groups in the abundance of p-DRP-1^Ser616^ (*p* = .437, Figure 2A), total DRP-1 (*p* = .274, Figure 2B), phospho/total DRP-1 (*p* = .744, Figure 2C), total MFF (*p* = .435, Figure 2E), phospho/total MFF (*p* = .950, Figure 2F), MFN-2 (*p* = .350, Figure 2J). We observed an interaction effect on the expression of phospho/total DRP-1 (*p* = .037, Figure 2C), with no effect of RET on any other markers of fission/fusion dynamics. Further posthoc analysis of phospho/total DRP-1 revealed a ∼54% increase in the 1h post-RET period in MA only (*p* = .035, Figure 2C).

### 3.3 Mitochondrial energy metabolism

**FIGURE 3:**
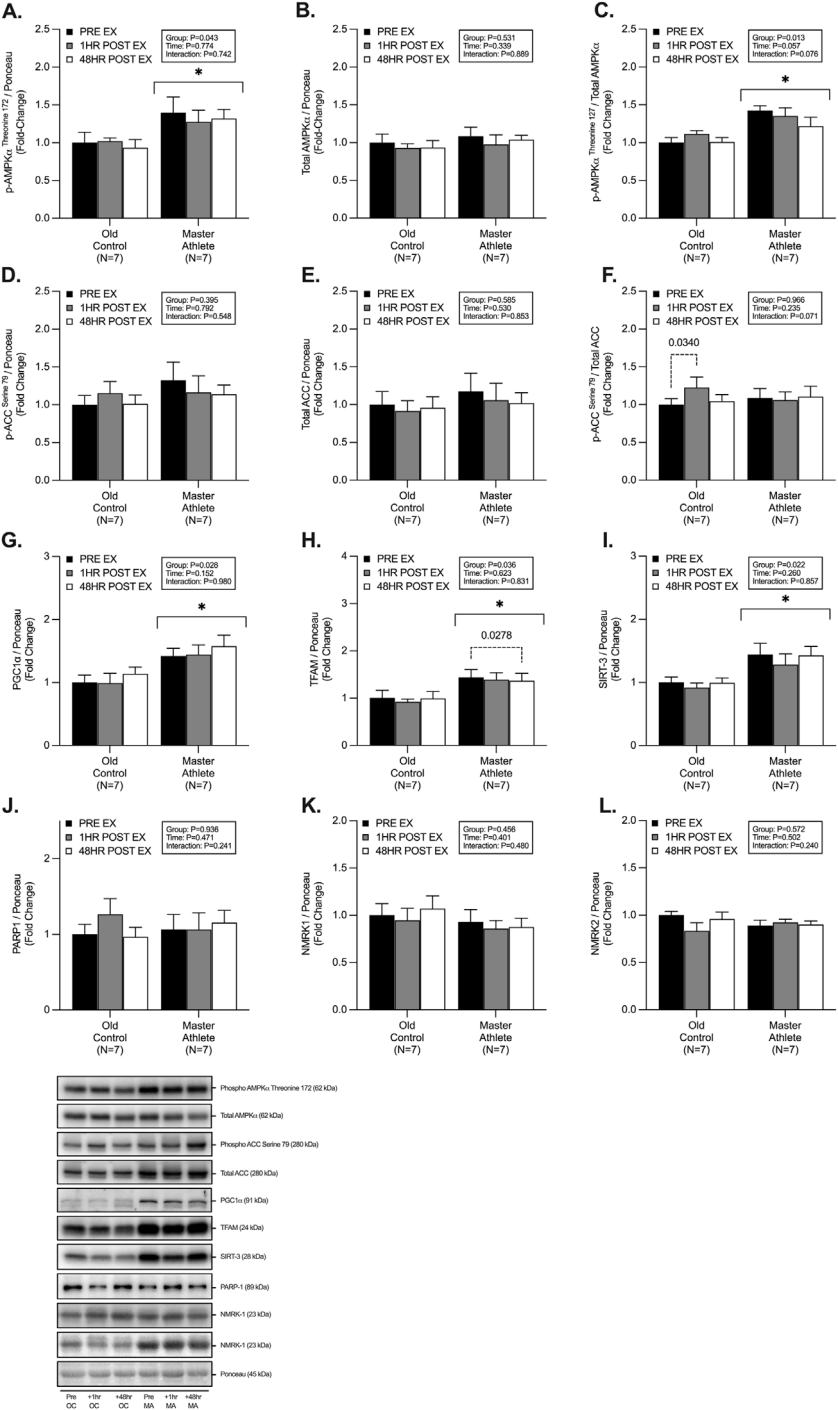
Master athletes display greater abundance of proteins involved in mitochondrial biosynthesis compared with old controls. Intracellular mitochondrial signalling of phosphorylated AMPKα^Threonine172^ **(A)** total AMPKα **(B)** phosphorylated:total AMPKα **(C)**, phosphorylated ACC^Serine79^ **(D)**, total ACC **(E)**, phosphorylated:total ACC **(F)**, PGC1α **(G)**, TFAM **(H)**, SIRT-3 **(I)**, PARP-1 **(J)**, NMRK1 **(K)**, and NMRK2 **(L)** at rest (black), 1 h post-resistance exercise (grey), and 48 h post-resistance exercise (white) in older untrained controls (n = 7) and master athletes (n = 7). all proteins are expressed relative to the total protein stain (ponceau s) and, where relevant, expressed at phosphorylated:total as a proxy for the respective activation status of the target protein. Significance was set at *p* < .05. The symbol “∗” with solid black bars indicates significantly different from older untrained control (*p* < .05). The *p*-value with dotted lines indicates significantly different from pre-exercise rest (*p* < .05). Values are presented as fold-change relative to pre-exercise old control.

We observed significant group differences between MA and OC, with MA displaying a greater abundance of; p-AMPKα^Thr172^ (∼37%, *p* = .043, Figure 3A), phospho/total AMPKα (∼28%, *p* = .013, Figure 3C), PGC1α (∼42%, *p* = .028, Figure 3G), TFAM (∼43%, *p* = .03, Figure 3H), and SIRT-3 (∼43%, *p* = .02, Figure 3I). We observed no differences in abundance of, total AMPKα (*p* = .531, Figure 3A), p-ACC^Ser79^ (*p* = .395, Figure 3D), total ACC (*p* = .585, Figure 3E), phospho/total ACC (*p* = .966, Figure 3F), PARP1 (*p* = .936, Figure 3J), NMRK1 (*p* = .456, Figure 3K) and NMRK2 (*p* = .572, Figure 3L) between groups. Post-hoc analysis revealed a small but significant 1h post-RET increase in phospho/total ACC in OC (∼22%, *p* = .034, Figure 3F), as well as a reduction in expression in TFAM in MA at 48h post-RET (∼5%, *p* = .027, Figure 3H).

## 4 Discussion

The contribution of skeletal muscle mitochondrial metabolism to the progression of sarcopenia is a highly debated topic with several authors reporting substantial declines in mitochondrial function with ageing (Pollock et al., 2018; Joanisse et al., 2020b). By contrast, endurance-trained MA cyclists display a greater abundance of mitochondrial-related proteins and show higher mRNA gene expression of MFN-2, ETC complexes, and PGC1α compared with untrained OC and YA (Pollock et al., 2018; Joanisse et al., 2020b). The current data support and extend on recent findings by Joanisse et al. (Joanisse et al., 2020b) by comprehensively assessing mitochondrial signalling at a protein-specific level in endurance-trained Master Athletes (MA) and untrained age-matched OC. This is particularly important because there are reported divergences between mRNA abundance (transcription) and the subsequent downstream changes in protein expression (translation) (Miller et al., 2016; Robinson et al., 2017c). In addition, we determined the responsiveness of mitochondrial protein expression during recovery from RET, a known potent stimulus for myofibrillar remodelling and muscle hypertrophy with unclear effects on mitochondrial signalling. We observed significantly greater CS activity (∼45%) and mitochondrial protein expression (OXPHOS, OPA-1, Mitofilin, PGC1α and SIRT-3) in MA compared with OC. Interestingly, we detected a significant decrease in CS activity (∼18%) in OC but not MA following RET. Furthermore, we demonstrated an upregulation of p-DRP-1^Ser616^ in MA at 1 h post-RET. However, we detected no other differences between OC and MA in the abundance of selected fission-fusion markers (MFN-2, DRP-1, MFF), energetic markers (AMPK, ACC, PGC1α, PARP1, NMRK1/2) at rest or following RET. Overall, these data show that MA maintain a greater abundance of mitochondrial-related protein expression compared with OC and that RET did not alter acute mitochondrial remodelling in the acute (1 h post-RET) and delayed (48 h post-RET) recovery period in OC and endurance-trained MA.

### 4.1 Mitochondrial content and volume

Intrinsic mitochondrial volume (assessed herein by CS activity) is a key determinant of skeletal muscle mitochondrial health and a validated marker of mitochondrial content (Larsen et al., 2012). Metabolic profiling of >140 adults (aged 19–89) has demonstrated a 5%–8% decline per year in CS protein content and activity (Short et al., 2005b). Here we report ∼45% and ∼74% higher resting CS activity and CS protein abundance in MA compared with OC. These data are similar to Gries et al., who report that CS activity in endurance-trained MA was ∼47% greater than OC and ∼4% greater than healthy, active YA (Gries et al., 2018). Increased CS activity has been reported following acute and chronic endurance/interval-based exercise (Vigelsø et al., 2014). However, the activity of CS is less well understood in models of RET, and to a lesser extent, in cohorts of older adults (Parry et al., 2020). Modest increases in CS activity (in YA and OC) have been reported with chronic RET (Tang et al., 2006; Ngo et al., 2012; Frank et al., 2016; Moro et al., 2019; Fritzen et al., 2020; Lamb et al., 2020), with others reporting no change (Parise et al., 2005a; 2005b; Groennebaek et al., 2018b; Gharahdaghi et al., 2019) or even a decline in CS activity (Haun et al., 2019). However, the lack of heterogeneity between RET studies (e.g., RET protocols and experimental approaches) may explain, at least in part, these equivocal observations. Specifically, due to an appreciable increase in myofibrillar and sarcoplasmic protein fractions following RET, mitochondrial density and/or content may appear unchanged, or reduced following a RET intervention (Macdougall et al., 1979), as this is not observed in models of endurance exercise when negligible hypertrophy is detected (Parry et al., 2020). To date, CS activity has not been established in the acute or delayed post-RET period in human skeletal muscle. Here, we observed a significant ∼18% decrease in CS activity in OC 48 h post-RET but no change in CS activity in MA. Interestingly, Ogborn et al. observed a ∼32% decline in CS mRNA following acute RET in untrained OC unaccustomed to RET (Ogborn et al., 2015). This decline may reflect a preferential upregulation in muscle protein anabolism to support tissue remodelling/recovery after a novel contractile stimulus, which may not be the case in long-term endurance-trained MA. Consistent with this, we previously observed a ∼9% increase in myofibrillar protein synthesis over the 48h post-RET in both OC and MA (McKendry et al., 2019). Therefore, further evidence is needed to determine the temporal changes in CS enzyme activity and other mitochondrial complexes with ageing and exercise training status.

The expression of proteins involved in oxidative phosphorylation within the ETC represent key markers of mitochondrial content and have previously been shown to be significantly decreased in OC compared with MA (Holloway et al., 2018a; Pollock et al., 2018; Joanisse et al., 2020b; Mesquita et al., 2020). Endurance exercise positively influences ETC protein expression; however, the acute and chronic impacts of RET on ETC protein adaptation remains inconclusive. Chronic RET interventions have been reported to increase ETC protein expression in older adults (Porter et al., 2015b; Holloway et al., 2018b; Mesquita et al., 2020). However, studies of the acute regulatory changes to these ETC proteins with RET are lacking. It is possible that acute changes in ETC expression may not be sensitive to RET (Ogborn et al., 2015; Mesquita et al., 2020) and/or the timing of muscle biopsy sampling has not been appropriate to detect any potential changes in ETC protein expression (Parry et al., 2020). In agreement with this notion, we did not observe any acute changes in ETC protein expression (McGinley and Bishop, 2016; Kuang et al., 2020). Despite detecting no effect of RET, we observed a significantly greater abundance of all the individual ETC complexes (I-V) in MA compared with OC. Moreover, combined ETC protein complexes were ∼83% greater in MA compared with OC, suggesting greater overall mitochondrial health. However, further detailed assessments of permeabilized skeletal muscle fibres *via* high-resolution respirometry are necessary to determine if the observed elevated CS activity ETC protein content in MA translate into greater respiratory function and bioenergetic health.

### 4.2 Mitochondrial fission and fusion

Mitochondrial quality control relies on several tightly orchestrated signalling proteins involved in the fission and fusion of damaged organelles to maintain cellular health, function, and muscle mass (Romanello et al., 2010; Romanello et al., 2019; Tezze et al., 2017a; Favaro et al., 2019a; Favaro et al., 2019b; Bell et al., 2019). Fission events are coordinated by AMPK-mediated phosphorylation of MFF^Ser146^ to act as a DRP-1 adaptor and allow the binding of DRP-1 to the outer mitochondrial membrane (Otera et al., 2010; Toyama et al., 2016). On the contrary, mitochondrial fusion is synchronized by MFN-2 and OPA-1 on the inner mitochondrial membrane (Tezze et al., 2017b; Romanello et al., 2019). Human data on fission-fusion dynamics suggests MA maintain greater expression of OPA1-, MFN-1, MFN-2, and DRP-1 compared with OC and is similar to that of YA (Tezze et al., 2017a; Moore et al., 2019). In the present study, whilst we did not observe greater expression of MFN-2 and DRP-1, we did detect a significantly greater abundance of OPA-1 (∼36%), FIS-1 (∼36%) and Mitofilin (∼133%) in MA. Interestingly, OPA-1 has recently been implicated in inner mitochondrial membrane cristae formation and remodelling and, therefore, represents a crucial marker of fusion dynamics and overall mitochondrial functioning (Frezza et al., 2006; Varanita et al., 2015). Greater expression of OPA-1 is also associated with muscle mass protection *in vivo* when transgenically overexpressed following denervation-induced atrophy (Varanita et al., 2015; Romanello et al., 2019). Further, when probing post-translational modifications of fission-fusion dynamics, we observed ∼15% greater expression of p-MFF^Ser146^ in MA compared with OC, with only the MA group demonstrating an ∼57% increase in post-RET activation of p-DRP-1^Ser616^. On the contrary, p-DRP-1^Ser616^ decreased by ∼32% in OC following RET. *In vivo* models have determined the critical role of DRP-1 following exercise and that muscle-specific knockdown/knockout results in blunted exercise adaptation, muscle atrophy, mitochondrial dysfunction and premature animal death (Favaro et al., 2019a; Dulac et al., 2020). Taken together, these data suggest that MA maintain greater skeletal muscle mitochondrial quality control mechanisms to more efficiently fragment damaged and defective organelles for degradation to maintain mitochondrial proteostasis, function and muscle mass.

### 4.3 Mitochondrial biosynthesis

RET can augment mitochondrial turnover and protein content (Holloway et al., 2018a; Mesquita et al., 2020). and, therefore, mitochondrial biosynthesis markers are also expected to increase with RET (Groennebaek and Vissing, 2017). Mitochondrial biogenesis is controlled by the transcription of nuclear and mitochondrial encoded genes, with PGC1α and TFAM being central regulators (Handschin and reviews, 2016). However, the role of PGC1α for training-induced mitochondrial biogenesis *in vivo* has recently been questioned (Rowe et al., 2012; Islam et al., 2020), with other markers being identified as critical regulators (e.g., Nrf2, ERRγ, PPARβ, LRP130) (Islam et al., 2020). Whilst endurance and high-intensity interval training (i.e., HIIT) exercise upregulates the PGC1α-TFAM pathway (Pilegaard et al., 2003), less is understood in models of RET. Herein, MA displayed a greater expression of p-AMPK^Thr172^, PCG1α, and TFAM compared with OC. However, in our study, RET was not sufficient to augment any increase in biosynthetic signalling. This is not entirely surprising as others have observed inconsistencies in the response of regulators of mitochondrial biogenesis following RET, with no changes in mRNA or protein, despite reported increases in mitochondrial protein turnover (Wilkinson et al., 2008; Burd et al., 2012; Robinson et al., 2017a). Interestingly, our cohort of MA displayed a ∼42% greater abundance of PGC1α protein expression compared with OC. In contrast with previous studies, the abundance of PGC1α protein expression in MA has been reported to be as much as ∼190% greater than OC and YA (Joanisse et al., 2020b), despite similar weekly exercise training history, training volume and phenotype to the current MA cohort. Others have observed a similarly greater abundance of PGC1α in “high” compared with “low” functioning older adults (Tezze et al., 2017a). Since PGC1α is a key signalling node in initiating mitochondrial biogenesis, the increased protein content may reflect the greater mitochondrial mass and content observed in the MA. Thus, mitochondrial biogenesis may be exercise and/or activity-dependent and not affected by ageing *per se*.

PGC1α also directly regulates sirtuin and NAD + -dependant signalling, such as SIRT-3, PARP1, and NMRK1/2. Here we observed that MA displayed greater expression (∼38%) of SIRT-3, similar to previously reported data (de Guia et al., 2019). However, we observed no differences in PARP1 or NMRK1/2 protein expression between groups. Interestingly, chronic RET has recently been reported to reverse age-related declines in NAD + salvage signalling, particularly NAMPT expression (de Guia et al., 2019; Lamb et al., 2020). Therefore, further investigation into tissue-specific and circulating NAD + metabolites, as previously undertaken in healthy older adults (Elhassan et al., 2019), warrants investigation in MA to determine if these populations maintain the NAD + metabolome and potential resistance to the age-associated decline in NAD + metabolites.

Although the present study provides important insights into the regulation of mitochondrial activity and bioenergetics, we acknowledge there are limitations. Our interpretation is limited by the timing of skeletal muscle biopsy sampling. In contrast, the time course in mRNA, protein expression, and phosphorylation status of mitochondrial signalling cascades has been extensively examined in the context of endurance and HIIT-based exercise (McGinley and Bishop, 2016; Kuang et al., 2020). Therefore, the present data adds to the paucity of data on RET-induced mitochondrial signalling regulation and suggests MA display greater mitochondrial signalling health compared with age-matched controls. Unfortunately, due to limited sample size (N = 7 per group), future studies should seek to explore MA’s in larger cohorts. Furthermore, the use of stable isotopic tracers (mitochondrial protein synthesis), high-resolution respirometry (mitochondrial respiration, ATP production and H_2_O_2_ emission) and detailed biochemical analysis of molecular signalling events post-RET are needed to improve our understanding of mitochondrial remodelling following single or repeated bouts of RET.

## 5 Conclusion

In summary, we observed greater CS activity and expression of proteins involved in mitochondrial ETC and quality control signalling in long-term endurance-trained master athletes compared with age-matched untrained individuals. However, mitochondrial protein expression and/or phosphorylation status remained largely unchanged in the early (1 h) and late (48 h) phase of recovery following an acute bout of RET. Collectively, these data suggest that participating in regular aerobic exercise training across adulthood may support mitochondrial health by bolstering mitochondrial enzyme activity and expression of proteins involved in ETC and fission-fusion dynamics. In contrast, RET has little effect on acute mitochondrial-related signalling or enzyme activity.

## Data Availability

The raw data supporting the conclusions of this article will be made available by the authors, without undue reservation.
